# Evaluation of Internet-Based Interventions on Waist Circumference Reduction: A Meta-Analysis

**DOI:** 10.2196/jmir.3921

**Published:** 2015-07-21

**Authors:** Dong-Chul Seo, Jingjing Niu

**Affiliations:** ^1^College of Health SciencesDepartment of Health Education and ManagementEwha Womans UniversitySeoulRepublic Of Korea; ^2^School of Public HealthDepartment of Applied Health ScienceIndiana UniversityBloomington, INUnited States

**Keywords:** waist circumference, obesity, adiposity, Internet, intervention studies

## Abstract

**Background:**

Internet-based interventions are more cost-effective than conventional interventions and can provide immediate, easy-to-access, and individually tailored support for behavior change. Waist circumference is a strong predictor of an increased risk for a host of diseases, such as hypertension, diabetes, and dyslipidemia, independent of body mass index. To date, no study has examined the effect of Internet-based lifestyle interventions on waist circumference change.

**Objective:**

This study aimed to systematically review the effect of Internet-based interventions on waist circumference change among adults.

**Methods:**

This meta-analysis reviewed randomized controlled trials (N=31 trials and 8442 participants) that used the Internet as a main intervention approach and reported changes in waist circumference.

**Results:**

Internet-based interventions showed a significant reduction in waist circumference (mean change –2.99 cm, 95% CI −3.68 to −2.30, I^2^=93.3%) and significantly better effects on waist circumference loss (mean loss 2.38 cm, 95% CI 1.61-3.25, I^2^=97.2%) than minimal interventions such as information-only groups. Meta-regression results showed that baseline waist circumference, gender, and the presence of social support in the intervention were significantly associated with waist circumference reduction.

**Conclusions:**

Internet-based interventions have a significant and promising effect on waist circumference change. Incorporating social support into an Internet-based intervention appears to be useful in reducing waist circumference. Considerable heterogeneity exists among the effects of Internet-based interventions. The design of an intervention may have a significant impact on the effectiveness of the intervention.

## Introduction

The prevalence of obesity has been increasing worldwide for approximately 50 years and has now become a global pandemic [1]. Lifestyle interventions balancing energy intake and energy expenditure have been suggested as effective tools to treat obesity and prevent obesity-related health burdens [2,3]. Internet-based interventions can provide immediate, easy-to-access, and individually tailored support for behavior change, which attract a large number of individuals, including the young or elderly, healthy, disabled or sick, and various ethnicities [4,5]. It has been reported that Internet-based lifestyle interventions can be as effective as phone- or person-based interventions in reducing body weight [6-8]. In contrast, Internet-based interventions are more cost-effective than conventional interventions [9-12].

Waist circumference, as a simple and effective measure of central obesity, is a strong predictor of an increased risk for hypertension, diabetes mellitus, dyslipidemia, metabolic syndrome, and coronary heart disease independent of body mass index (BMI) [13,14]. Changes in waist circumference in response to lifestyle interventions reflect changes in central obesity [15,16]. Studies have reported that waist circumference can be reduced while no significant changes in body weight occur [17-19]. Few studies, however, have systemically evaluated the effect of lifestyle interventions on waist circumference change. Therefore, this study examined the effect of Internet-based lifestyle interventions on waist circumference change.

Previous reviews have reported that Internet-based interventions can promote physical activity and significantly reduce body weight [2,20,21]. Khaylis et al [22] conducted a systematic review of efficacious technology-based weight-loss interventions and identified self-monitoring, counselor feedback and communication, social support, structured programs, and individually tailored programs as a key to successful interventions. In addition, the literature identified goal setting, motivational interviewing, and incentives as potential factors that increase intervention effectiveness [9,11,23-26]. Seo and Sa [27] also reported that the number of components was associated with the effect of lifestyle interventions. Based on the existing evidence, we hypothesize the following: (1) an Internet-based intervention can significantly reduce waist circumference; (2) Internet-based interventions reduce waist circumference more than conventional minimal interventions, such as those with usual care or information-only delivery; and (3) the number and type of components in lifestyle interventions are significantly associated with the effect on waist circumference change.

## Methods

### Search Strategy

An electronic search was performed in the following databases: Academic Search Premier, CINAHL Plus with Full Text, Educational Resource Information Center (ERIC), Health Source Nursing/Academic Edition, MEDLINE, PsycARTICLES, SPORTDiscus with Full Text Results, and ProQuest Dissertations and Theses A&I database. The search terms used various combinations of the following keywords or phrases: adiposity, weight, overweight, obese, obesity, lifestyle, nutrition, diet, intake, physical activity, exercise, eHealth, Web, online, email, electronic mail, Internet, social networking, treatment, therapy, interventions, management, trial, waist, central adiposity, random, control, and randomized controlled trial (RCT). After excluding ineligible studies, a manual search was conducted by screening the references of the remaining articles and contacting experts. The detailed search strategy can be found in Multimedia Appendix 1.

### Inclusion Criteria

Studies were selected if they met all of the following criteria: (1) published in English peer-reviewed journals between 1980 and April 2014 or dissertations/theses written in English that reported relevant yet unpublished results and were uploaded before April 2014, (2) studies based on RCTs, (3) studies that used the Internet as a major intervention tool in at least 1 arm, (4) studies that used lifestyle interventions (which promote healthy diet, physical activity, or both), (5) studies that reported the mean and standard deviation (SD) or standard error (SE) of the waist circumference, and (6) studies involving adults (aged ≥18 years). Studies were excluded if special diets or medications were used in the intervention or only follow-up data of an intervention were reported.

### Data Extraction

The following data were extracted from each included study and substudy: (1) general information, such as the name of the first author and year of publication/completion; (2) characteristics of the substudy, such as intervention location, number of participants, intervention length, frequency, retention rate, participants’ compliance, features of the intervention arm, approaches used in adjunction to the Internet (eg, personal contacts via phone, in-person visits, or other devices), intervention content, and whether or not theory, tailoring, self-monitoring and feedback on performance, goal setting, motivational interviewing, social support/social change, and incentives for weight loss were used in the intervention; (3) characteristics of the participants, such as general obesity status, reported existing diseases, mean age, and percentage of male participants; and (4) the mean and SD or SE of the waist circumference at baseline and immediately after the intervention, and the waist circumference change. The SE of the waist circumference change was calculated using the baseline and follow-up SD or SE, assuming an intracorrelation coefficient of 0.5 between pretest and posttest [28], when the SD or SE of the waist circumference change was not reported. The intention-to-treat analysis results were extracted and used when available. The risk of bias was assessed using the Cochrane Collaboration tool [29] and this assessment was used to guide the interpretation of study results.

### Data Analysis

Each reported arm was treated as an independent substudy. Treatments that were unlikely to have effects on waist circumference change, such as no intervention, delayed intervention, usual care, and information-only groups, were categorized as “minimal interventions.” Paper-, phone-, and person-based interventions were grouped together as “other interventions” because only 6 trials used any of these interventions. We calculated the overall effect sizes of waist circumference changes in Internet-based, minimal, and other interventions. Next, we compared the effects between Internet-based interventions with minimal or other interventions. To examine the effect of a “unique intervention component” on waist circumference changes, intervention components were coded as 0 for the component delivered in both conditions, 1 for the unique component in Internet-based intervention, and 2 for the unique component in minimal intervention. The number of times each intervention component was uniquely found only in Internet-based interventions was computed.

Effect sizes were presented as the mean waist circumference change in centimeters with a 95% confidence interval (CI). Funnel plot and Begg’s test were used to test publication bias. The I^2^ index was used to test between-study heterogeneity. A meta-regression was performed to identify characteristics that were significantly associated with differences in waist circumference changes between Internet-based interventions and minimal interventions, although it was likely underpowered. Due, in part, to the concern about the possible insignificant findings arising from low power, another meta-regression was performed for the waist circumference changes from baseline to posttest. The adjusted *R* ^*2*^ was calculated to present the predictive power of meta-regression models. Random effects models were used if significant between-study heterogeneity was detected. Analyses were performed using Stata 13 (StataCorp LP, College Station, TX, USA).

## Results

### Overview

**Figure 1:**
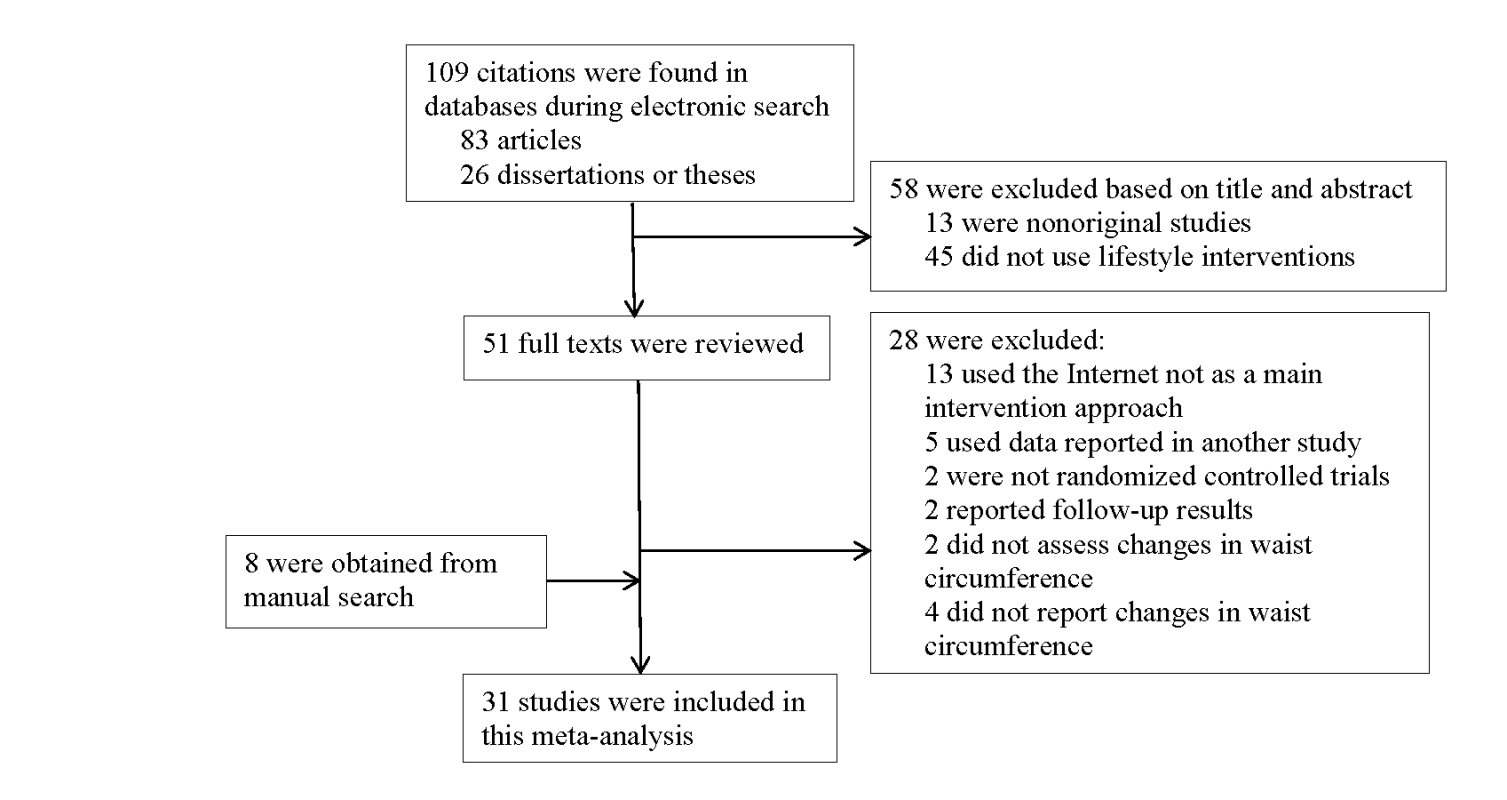
Flow chart of literature search.

After removing duplicates, the electronic search retrieved 83 articles and 26 dissertations or theses. The manual search retrieved 8 additional studies. Four studies indicated measurement of waist circumference, yet failed to report adequate information on the waist circumference. We contacted the authors but could not obtain additional information necessary for meta-analysis. Thus, these 4 studies were excluded from analysis. Figure 1 demonstrates the flow of the literature search. The studies were reviewed independently by 2 reviewers and any disagreement encountered was resolved by discussion.

### Characteristics of Included Studies

This review includes 31 intervention trials involving 72 intervention arms and 8442 adults [6-12,23,25,30-51]. The number of total participants ranged from 21 to 1692, and the mean sample size was 272 per study. Four studies only recruited women and 5 studies only recruited men. Fifteen of 31 interventions were conducted in the United States. Among substudy participants, the mean age ranged from 19.0 to 64.9 years and the mean baseline waist circumference ranged from 81.7 to 128.4 cm. The intervention length ranged from 4 weeks to 2 years. The retention rate ranged from 21.7% to 100%, and the mean retention was 75.2%. Characteristics of the included substudies are provided in Table 1.

**Table 1:** Descriptive data of substudies included in this meta-analysis (N=72).

Author	Year	Arm	Features	N	Waist circumference (cm)		
					Baseline	Post	Δ Mean (SD)
Bennett [9]	2010	Minimal^a^	Brochure	50	N/A	N/A	–1.9 (10.8)
		Internet	Online program + forum	51	N/A	N/A	–1.9 (10.8)
Bischoff [30]	2010	Basic Internet-based	Online program + email contacts	22	94.7	92.6	–2.1 (3.5)
		Enhanced Internet-based	Basic + goal setting	21	83.8	82.5	–1.3 (1.9)
Booth [10]	2008	Basic Internet-based	Online PA program + forum + email feedbacks	26	96.9	N/A	–4.5 (4.5)
		Enhanced Internet-based	Basic + nutrition component	27	95.6	N/A	–3.2 (2.9)
Bukhari [31]	2009	Minimal^a^	One class + a counseling	11	98.8	100.6	1.8 (16.9)
		Internet	Online programs	16	107.5	101.8	–5.7 (14.6)
Carr [32]	2008	Minimal^a^	None	18	99.2	99.8	0.6 (2.1)
		Internet	Online sessions + email contacts	14	100.6	96.6	–4.0 (2.5)
Chambliss [33]	2011	Minimal^a^	None	28	100.1	N/A	–0.6 (5.2)
		Basic Internet-based	Online monitor + email counseling	33	97.1	N/A	–3.4 (4.6)
		Enhanced Internet-based	Basic + behaviorally tailored	34	98.2	N/A	–2.8 (5.4)
Chen [34]	2013	Minimal^a^	None	31	88.9	88.3	–0.6 (10.2)
		Internet	Online program + feedbacks	32	91.9	88.4	–3.5 (11.1)
Chung [35]	2014	Minimal^a^	None	19	94.5	92.6	–1.9 (8.3)
		Paper	Logbook	16	93.2	89.1	–4.1 (7.1)
		Internet	Online logs + evaluation	19	91.9	88.5	–3.4 (11.0)
Collins [36]	2012	Minimal^a^	None	104	107.2	N/A	0.3 (3.1)
		Basic Internet-based	Online programs + forums + email contacts	99	106.9	N/A	–2.6 (4.0)
		Enhanced Internet-based	Basic + personalized + feedbacks	106	106.6	N/A	–3.2 (5.0)
Dekkers [37]	2011	Minimal^a^	None	49	101.7	99.2	–2.5 (8.9)
		Phone	Phone sessions + counseling	44	99.9	96.4	–3.5 (10.6)
		Internet	Online sessions + email counseling	48	102.9	99.4	–3.5 (11.3)
Hansen [38]	2012	Minimal^a^	None	585	89.6	89.1	–0.5 (8.4)
		Internet	Online program + forum	583	90.1	90.0	–0.1 (8.5)
Herrick [39]	2009	Minimal	None	860	81.7	N/A	0.7 (4.7)
		Internet	Online programs + email reminders	832	81.9	N/A	0.3 (2.9)
Hunter [40]	2008	Minimal^a^	None	222	94.2	93.4	–0.4 (3.8)
		Internet	Online program + feedbacks	224	94.5	92.2	–2.1 (4.3)
Kang [41]	2010	Minimal^a^	General information	75	85.4	88.6	3.2 (8.8)
		Basic Internet-based	1-year email education	25	83.2	84.3	1.1 (5.5)
		Enhanced Internet-based	2-year email education	25	89.1	87.3	–1.8 (3.0)
Mehring [23]	2013	Minimal^a^	Usual care	77	110.9	104.4	–6.9 (6.9)
		Internet	Online programs	109	107.3	106.6	–2.4 (5.0)
Mobley [42]	2006	Basic in-person	In-person counseling	32	100.7	99.3	–1.4 (9.1)
		Enhanced in-person	In-person counseling	33	101.8	98.5	–3.3 (9.7)
		Basic Internet-based	Online programs	29	102.7	102.4	–0.3 (10.1)
		Enhanced Internet-based	Online programs	29	101.6	103.4	1.8 (8.4)
Morgan [11]	2009	Minimal^a^	None	31	102.8	N/A	–5.2 (5.4)
		Internet	Online programs + notice board + email feedbacks	34	103.4	N/A	–4.4 (5.7)
Morgan [6]	2011	Minimal	None	45	99.4	N/A	1.5 (4.5)
		Internet	Online program + email feedbacks	65	101.6	N/A	–4.4 (4.8)
Morgan [43]	2013	Minimal	None	52	113.6	N/A	–0.8 (2.9)
		Paper	Books	54	112.6	N/A	–3.7 (4.5)
		Internet	Online programs + email feedbacks	53	113.7	N/A	–5.4 (5.2)
Patrick [12]	2011	Minimal^a^	Website with general health information	217	112.9	111.6	–1.3 (11.4)
		Internet	Online assessment + sessions + feedbacks + email counseling	224	113.7	112.1	–1.6 (11.4)
Pressler [44]	2010	Basic Internet-based	Nonstructured	27	101.9	98.3	–3.6 (8.6)
		Enhanced Internet-based	Structured	50	100.5	98.0	–2.5 (7.8)
Pullen [45]	2008	Basic Internet-based	Online program	8	98.1	96.2	–1.9 (6.5)
		Enhanced Internet-based	Basic + online discussions	8	91.1	85.7	–5.4 (6.4)
Rogers [7]	2012	Paper	Group sessions	14	125.9	121.9	–4.0 (8.1)
		Basic Internet-based	Online programs	12	128.4	121.6	–6.8 (9.3)
		Enhanced Internet-based	Basic + Bluetooth	13	126.3	120.1	–6.2 (12.6)
Seely [46]	2013	Minimal^a^	None	13	N/A	N/A	–2.8 (2.3)
		Internet	Facebook support group	11	N/A	N/A	–2.9 (3.0)
Tate [47]	2001	Basic Internet-based	Online program + email reminders	45	98.4	N/A	–3.1 (4.4)
		Enhanced Internet-based	Basic + online behavioral therapy	46	98.5	N/A	–6.4 (5.5)
Tate [48]	2003	Basic Internet-based	Online program + email reminders	46	111.0	N/A	–4.4 (5.7)
		Enhanced Internet-based	Basic + email counseling	46	108.0	N/A	–7.2 (7.5)
van Genugten [49]	2012	Basic Internet-based	General information online	239	95.7	93.2	–2.5 (8.6)
		Enhanced Internet-based	Basic + computer tailored + forum	241	95.9	94.4	–1.5 (9.7)
van Wier [8]	2009	Minimal^a^	Brochure	231	101.5	99.5	–2.0 (9.9)
		Internet	Online program + email counseling	236	102.6	98.6	–1.9 (6.3)
		Phone	Phone counseling	235	101.5	98.2	–1.2 (11.7)
Webber [50]	2010	Basic Internet-based	Online program + message board	36	96.5	N/A	–3.6 (5.2)
		Enhanced Internet-based	Basic + motivational treatment	34	97.3	N/A	–3.6 (5.2)
Wijsman [25]	2013	Minimal^a^	None	112	101.4	N/A	–1.3 (3.6)
		Internet	Online program + email counseling	114	102.3	N/A	–2.3 (3.8)
Yoo [51]	2009	Minimal^a^	None	54	91.3	89.1	–2.2 (7.5)
		Internet	Online monitoring + feedbacks	57	89.5	86.8	–2.7 (9.8)

^a^ Minimal arm includes control, wait-list, usual care groups or the group that only received standard health information.

Of the 72 intervention arms reviewed in the current study, 33 adapted behavioral theories or therapy principles, 40 prompted self-monitoring of behavior, 39 used feedback on performance and individual tailoring or counseling, 31 used goal setting, 15 planned online social support/social change, 6 used motivational interviewing, and 2 used incentives to encourage weight loss. Regarding the total number of components used in each arm, approximately one-third of the arms used none, one-third used 1 to 3 components, and the final one-third used 4 to 6 components. Details about the arm components can be found in Multimedia Appendix 2. A total of 24 pairs were extracted for comparison between Internet-based interventions and minimal interventions. The number of times each component was uniquely found only in Internet-based interventions was 16 for theory, 21 for tailoring, 21 for monitoring, 15 for goal setting, 4 for motivational interviewing, 11 for social support, and 1 for incentive.

Bias assessment showed the following results: 17 studies provided details on random sequence generation, 18 studies provided details on allocation concealment, only 2 studies reported blinding participants, and 9 studies reported blinding assessors. As shown in Multimedia Appendix 3, the bias assessment indicated no evidence of selective reporting of outcomes. As shown in the funnel plot for publication bias (see Multimedia Appendix 4), no significant publication bias was detected (*P*=.31 for Begg’s test) for Internet-based interventions as evaluated by the waist circumference change in each study arm. Multimedia Appendix 5 shows content and supplementary approaches of substudies included in this meta-analysis.

### Overall Effects of Interventions

**Figure 2:**
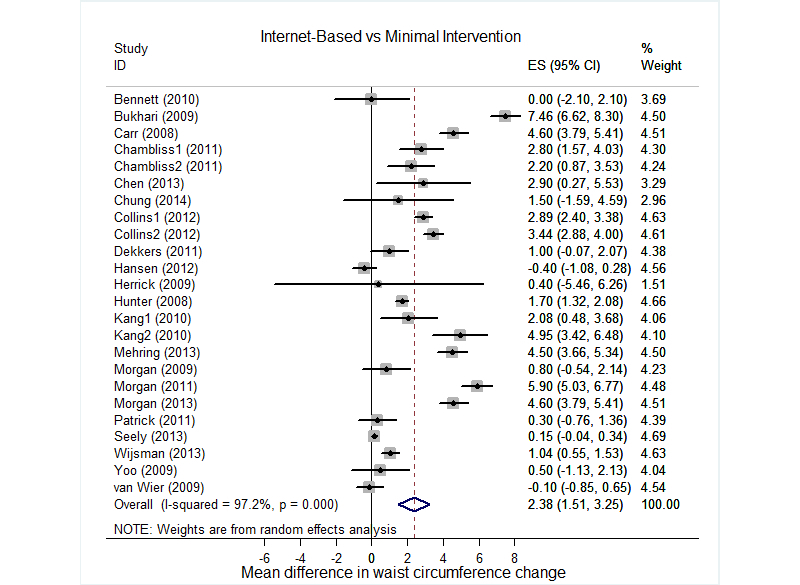
Forest plot for the differences in waist circumference changes between Internet-based interventions and minimal interventions. % Weight: weights assigned to substudies.

**Figure 3:**
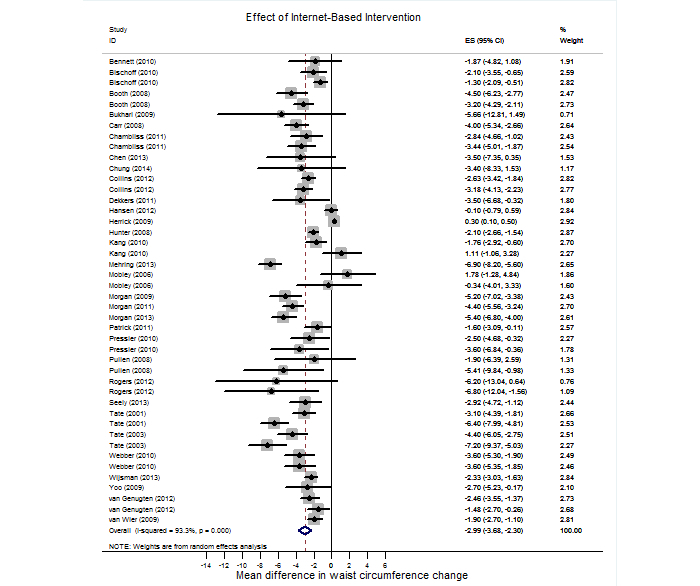
Forest plot for the effect of Internet-based intervention on waist circumference changes. % Weight: weights assigned to substudies.

Figure 2 shows the differences in waist circumference change between Internet-based interventions and minimal interventions. Internet-based interventions showed significantly better effects on waist circumference reduction (mean change 2.38 cm, 95% CI 1.51-3.25) compared with minimal interventions. Few differences were observed with respect to the waist circumference change between Internet-based interventions and paper-, phone-, or person-based interventions (mean change −0.61 cm, 95% CI −2.05 to 0.83, *P=*.42). Figure 3 provides a forest plot representing the effect size of Internet-based interventions on the waist circumference change. Overall, Internet-based interventions significantly reduced the waist circumference (mean change −2.99 cm, 95% CI −3.68 to −2.30), whereas minimal interventions (mean change −0.81 cm, 95% CI −1.41 to −0.20) and other interventions (mean −2.82 cm, 95% CI −3.89 to −1.74) also reduced waist circumference. Large and significant between-study heterogeneity was observed (I^2^=93.3%, *P*<.001).

### Characteristics Associated With the Waist Circumference Change

The meta-regression of differences in waist circumference changes (changes in minimal intervention groups minus changes in Internet-based intervention groups) showed no significant associations between effect sizes with the content and number of unique intervention components. To further investigate the effect of intervention components, a meta-regression of waist circumference changes from baseline to posttest was conducted. Results of this meta-regression are shown in Table 2. Stepwise meta-regression showed that only the mean waist circumference at baseline (coefficient = −0.16, *P*<.001) and proportion of male participants (coefficient = −0.02, *P*=.02) were significantly associated with the effect on waist circumference change (I^2^=69.8%, *P*<.001) among the characteristics of the participants that included mean age, reported existing diseases, and status of general obesity. The waist circumference at baseline alone explained 45.3% of the between-study variation in waist circumference changes. Controlling for the baseline waist circumference and proportion of male participants (*R* ^*2*^=.58), the component of social support in Internet-based intervention was associated with a significantly better effect on waist circumference changes (mean difference −1.16 cm, *P*=.03), which increased the *R* ^*2*^ to .66.

**Table 2:** Meta-regression of waist circumference change from baseline to posttest.^a^

Intervention component	Coefficient	SE	*P*	I^2^
Mean waist circumference at baseline (cm)	–0.16	0.03	<.001	69.8%
Male participants (%)	0.02	0.01	.02	
Length (weeks)^b^	0.01	0.01	.89	69.9%
Frequency (week per contact)^b^	–0.13	0.20	.54	71.4%
Retention (%)^b^	–0.02	0.01	.15	71.1%
Intervention content^b^				70.6%
Physical activity vs both	0.34	0.58	.56	
Nutrition vs both	–1.38	2.27	.55	
Theory (yes vs no)^b^	0.38	0.53	.48	69.7%
Tailoring (yes vs no)^b^	–0.45	0.58	.45	69.9%
Monitoring (yes vs no)^b^	–0.62	0.66	.35	69.6%
Goal setting (yes vs no) ^b^	0.50	0.57	.38	69.6%
Motivational interviewing (yes vs no) ^b^	–0.09	0.77	.91	69.5%
Social support (yes vs no)^b^	–1.16	0.51	.03	66.9%
Number of components^b^	–0.12	0.16	.48	69.6%

^a^ The incentive variable was not included in the model because only 1 study used incentives to encourage weight loss.

^b^ Controlled for mean waist circumference at baseline and percentage of male participants.

## Discussion

This study was the first attempt to the authors’ knowledge that evaluated effect sizes of Internet-based lifestyle interventions on decreasing waist circumference. This meta-analysis showed that Internet-based interventions not only decreased waist circumference substantially at posttest (a mean decrease of 2.99 cm), but also did so significantly more than minimal interventions. Given that a meta-analysis of workplace physical activity and dietary behavioral interventions only demonstrated an average waist circumference reduction of 0.67 cm (95% CI −1.96 to 0.63) [52] and another meta-analysis of antiobesity drugs showed an additional waist circumference reduction of 1.72 to 3.58 cm at 3 months among overweight or obese adults compared with standard care groups [53], Internet-based interventions appear to have a promising effect on waist circumference reduction. It deserves mention that this meta-analysis found few differences in waist circumference reduction between Internet-based intervention and paper-, phone-, or person-based lifestyle interventions. Considering that Internet-based interventions can attract a larger number of individuals with various backgrounds [2,4,54], can provide immediate and easy-access support at a lower cost [21,55], are more accessible to older adults and residents of geographically isolated communities [6,11,26], and are less obtrusive [26] than traditional methods, the substantial effect of Internet-based lifestyle interventions on the waist circumference change found in this study adds to the reason that Internet-based rather than traditional lifestyle interventions should be more widely and boldly explored.

Of the 31 trials reviewed in this study, 24 differences between Internet-based intervention and minimal intervention were identified. Compared with minimal interventions, Internet-based interventions included 1 to 6 unique intervention components. In addition, 13 trials tested the differences between basic and enhanced Internet-based interventions. Compared with the basic interventions, the enhanced intervention included 1 or 2 additional intervention features, such as adding healthy diet promotion to physical activity promotion or adding Bluetooth technology to the basic intervention. As indicated in Table 1, some enhanced interventions had a better effect on waist circumference change whereas others had a worse effect than basic interventions. The result of this study indicates that no conclusive evaluation is warranted on the efficacy of such additional features of the Internet-based lifestyle interventions.

To complement findings drawn from current and previous systematic reviews, we tested the associations between key intervention characteristics and waist circumference reduction in meta-regression models where independent variables were selected based on previous findings [9,12,22,27]. We examined whether the content and number of unique intervention components could adequately predict the differences in waist circumference changes between Internet-based interventions and minimal interventions only to find no significant association. The lack of significant association may be explained by lack of power due to the small sample size (n=24). It is also possible that the effect size can be explained by intervention features not tested in the current study. Similar future meta-analysis research is warranted that includes more studies and possibly a different framework. Due, in part, to the concern about the possible insignificant findings arising from low power, another meta-regression was performed for the waist circumference changes from baseline to posttest. We found that only the availability of social support was significantly associated with the waist circumference change after controlling for the main characteristics of participants. This means that providing sufficient social support is important to improve the efficacy of Internet-based lifestyle interventions. The lack of significant associations between waist circumference reduction and intervention length, intervention topic (nutrition only, physical activity only, or both), and the approach used in adjunction to the Internet (eg, personal contacts via phone, in-person visits, or use of such devices as Bluetooth, pedometer, or accelerometer) deserves further research. Although other intervention characteristics did not yield significant results in this review, further investigations are needed to draw conclusive suggestions.

It is worth noting that considerable heterogeneity remained after controlling for baseline waist circumference, gender, and intervention components identified by this study. It indicates that there is heterogeneity in effect sizes among the Internet-based interventions examined in this review that has yet to be accounted for. This may have to do with lack of frameworks that informed the design of Internet-based interventions reviewed in this study or lack of use of well-defined constructs or concepts. Previous studies have found that there is a lack of framework for the design of technology-based behavioral interventions and each research team used their own ways to develop and report technology-based interventions [56,57]. As a result, many of such technology-based intervention features lack comparability between different studies. Eysenbach and colleagues [58] developed the CONSORT-EHEALTH to standardize reports of eHealth/mHealth interventions, which has been very helpful in disseminating and comparing research reports. Recently, Schueller et al [56] proposed the modular system Purple to assist the development of Internet-based and mobile-based applications for health behavior change and Mohr et al [57] proposed a comprehensive framework, the Behavioral Intervention Technology (BIT) Model. These recently proposed frameworks and models should be fully utilized to inform the design of future technology-based interventions. In addition, for comparability and clarity of findings in behavior change interventions, it is desirable to use well-defined terms such as those shown in the Coventry, Aberdeen & London-Refined (CALO-RE) taxonomy [59]. These new frameworks and taxonomy of behavior change techniques will help increase comparability between different technology-based behavioral intervention studies as well as enhance the effectiveness of such interventions.

This meta-analysis review has the following limitations. First, gender-specific analyses were not performed due to a lack of gender-specific information, although baseline waist circumference and changes in the waist circumference may differ by gender. Future studies would be desirable that investigate gender-specific waist circumference changes of Internet-based interventions. Second, paper-, phone-, and person-based interventions were grouped together in this analysis due to a lack of data. Future research can be conducted to compare each mode of lifestyle interventions with other modes of interventions in terms of effect size when the sample size is appropriate. Third, the effect of compliance and incentives were not investigated in this study due to a lack of such information in the reviewed studies. Researchers are recommended to report information on participant compliance and incentives. Finally, considerable heterogeneity in waist circumference changes remained after controlling for covariates including baseline waist circumference and gender. This might indicate that reviewed interventions lacked frameworks that informed their study design. Thus, it is possible that not all the efficacious intervention components in reducing waist circumference for Internet-based lifestyle interventions might have been examined and analyzed.

In summary, Internet-based lifestyle interventions showed a significant and substantial effect on waist circumference change. Internet-based interventions showed comparable effects on the waist circumference change to paper-, phone-, and person-based interventions. Online social support appears to strengthen the effect of Internet-based programs on waist circumference reduction. Internet-based programs are recommended for obesity or lifestyle as effective and efficient interventions. It is also recommended to integrate online social support into Internet-based programs to achieve better effects on weight control.

## Multimedia Appendix 1

Search strategy.

## Multimedia Appendix 2

Components of sub-studies included in this meta-analysis.

## Multimedia Appendix 3

Table for the risk of bias assessment.

## Multimedia Appendix 4

Funnel plot for publication bias.

## Multimedia Appendix 5

Content and supplementary approaches of substudies included in this meta-analysis.

## Abbreviations

BIT: Behavioral Intervention Technology
ERIC: Educational Resource Information Center
RCT: randomized controlled trial
