# Supplementary material for: Evaluation of Internet-Based Interventions on Waist Circumference Reduction: A Meta-Analysis
Source: J Med Internet Res. 2015 Jul 21;17(7):e181. doi: 10.2196/jmir.3921 (PMC4527011; doi:10.2196/jmir.3921)
Supplement: Supplementary file 1 [file jmir_v17i7e181_app1.pdf]

Appendix 1: Search strategy

1) adiposity

2) weight

3) overweight

4) obese

5) obesity

6) lifestyle

7) nutrition

8) diet

9) intake

10) physical activity

11) exercise

12) ab(1 or 2 or 3 or 4 or 5 or 6 or 7 or 8 or 9 or 10 or 11)

13) eHealth

14) web

15) online

16) email

17) electronic mail

18) Internet

19) social networking

20) ab(13 or 14 or 15 or 16 or 17 or 18 or 19)

21) treatment

22) therapy

23) interventions

24) management

25) trial

26) ab(21 or 22 or 23 or 24 or 25)

27) waist

28) central adiposity

29) ab(27 or 28)

30) random

31) control

32) RCT

33) Ab(30 or 31 or 32)

34) review

35) meta

36) ti(34 or 35)

37) 12 and 20 and 26 and 29 and 33 not 36
