# Supplementary material for: Evaluation of Internet-Based Interventions on Waist Circumference Reduction: A Meta-Analysis
Source: J Med Internet Res. 2015 Jul 21;17(7):e181. doi: 10.2196/jmir.3921 (PMC4527011; doi:10.2196/jmir.3921)
Supplement: Supplementary file 2 [file jmir_v17i7e181_app2.pdf]

Appendix 2: Components of sub-studies included in this meta-analysis

Author	Year	Arm	Theory	Tailor	Monitor	Goal	Motivate	Social	Incentive	# Components
Bennett	2010	Minimal	No	No	No	No	No	No	No	0
		Internet	No	Yes	Yes	Yes	Yes	Yes	No	5
Bischoff	2010	Internet_b	Yes	Yes	Yes	No	No	No	No	3
		Internet_e	Yes	Yes	Yes	Yes	No	No	No	4
Booth	2008	Internet_b	Yes	Yes	Yes	Yes	No	Yes	No	5
		Internet_e	Yes	Yes	Yes	Yes	No	Yes	No	5
Bukhari	2009	Minimal	No	No	No	No	No	No	No	0
		Internet	Yes	No	No	No	No	No	No	1
Carr	2008	Minimal	No	No	No	No	No	No	No	0
		Internet	Yes	Yes	Yes	No	No	No	No	3
Chambliss	2011	Minimal	No	No	No	No	No	No	No	0
		Internet_b	No	Yes	Yes	No	No	No	No	2
		Internet_e	No	Yes	Yes	No	No	No	No	2
Chen	2013	Minimal	No	No	No	No	No	No	No	0
		Internet	No	Yes	Yes	No	No	No	No	2
Chung	2014	Minimal	No	No	No	No	No	No	No	0
		Paper	No	Yes	Yes	No	No	No	No	2
		Internet	No	Yes	Yes	No	No	No	No	2
Collins	2012	Minimal	No	No	No	No	No	No	No	0
		Internet_b	Yes	Yes	Yes	Yes	No	Yes	No	5
		Internet_e	Yes	Yes	Yes	Yes	No	Yes	No	5
Dekkers	2011	Minimal	No	No	No	No	No	No	No	0
		Phone	Yes	Yes	Yes	Yes	No	No	No	4
		Internet	Yes	Yes	Yes	Yes	No	No	No	4
Hansen	2012	Minimal	No	No	No	No	No	No	No	0
		Internet	Yes	Yes	Yes	Yes	No	Yes	No	5
Herrick	2009	Minimal	No	No	No	No	No	No	No	0
		Internet	Yes	No	No	Yes	No	No	No	2

Hunter	2008	Minimal	No	No	No	No	No	No	No	0
		Internet	No	Yes	Yes	No	Yes	No	No	3
Kang	2010	Minimal	No	No	No	No	No	No	No	0
		Internet_b	Yes	Yes	Yes	Yes	Yes	Yes	No	6
		Internet_e	Yes	Yes	Yes	Yes	Yes	Yes	No	6
Mehring	2013	Minimal	No	No	No	No	No	No	No	0
		Internet	Yes	Yes	Yes	Yes	No	Yes	No	5
Mobley	2006	Person_b	No	Yes	No	No	No	No	No	1
		Person_e	Yes	Yes	No	No	No	No	No	2
		Internet_b	No	Yes	Yes	Yes	No	No	No	3
		Internet_e	Yes	Yes	Yes	Yes	No	No	No	4
Morgan	2009	Minimal	No	No	No	No	No	No	No	0
		Internet	Yes	Yes	Yes	Yes	No	Yes	No	5
Morgan	2011	Minimal	No	No	No	No	No	No	No	0
		Internet	Yes	Yes	Yes	Yes	No	Yes	Yes	6
Morgan	2013	Minimal	No	No	No	No	No	No	No	0
		Paper	Yes	No	No	No	No	No	No	1
		Internet	Yes	Yes	Yes	Yes	No	Yes	No	5
Patrick	2011	Minimal	No	No	No	No	No	No	No	0
		Internet	Yes	Yes	Yes	Yes	No	No	No	4
Pressler	2010	Internet_b	No	No	No	No	No	No	No	0
		Internet_e	No	Yes	Yes	Yes	No	No	No	3
Pullen	2008	Internet_b	Yes	No	Yes	Yes	No	No	No	3
		Internet_e	Yes	No	Yes	Yes	No	Yes	No	4
Rogers	2012	Paper	Yes	Yes	Yes	Yes	No	No	No	4
		Internet_b	Yes	Yes	Yes	Yes	No	No	No	4
		Internet_e	Yes	Yes	Yes	Yes	No	No	No	4
Seely	2013	Minimal	No	No	No	Yes	No	No	No	1
		Internet	No	No	No	Yes	No	Yes	No	2
Tate	2003	Internet_b	No	No	No	No	No	No	No	0
		Internet_e	No	Yes	Yes	No	No	No	No	2

Tate	2001	Internet_b	No	No	No	No	No	No	No	0
		Internet_e	No	Yes	Yes	No	No	No	No	2
van Genugten	2012	Internet_b	No	No	No	No	No	No	No	0
		Internet_e	Yes	Yes	Yes	Yes	No	Yes	No	5
van Wier	2009	Minimal	No	No	No	No	No	No	No	0
		Internet	Yes	Yes	Yes	Yes	No	No	No	4
		Phone	Yes	Yes	Yes	Yes	No	No	No	4
Webber	2010	Internet_b	Yes	No	Yes	No	No	No	No	2
		Internet_e	Yes	No	Yes	Yes	Yes	No	No	4
Wijsman	2013	Minimal	No	No	No	No	No	No	No	0
		Internet	Yes	Yes	Yes	Yes	No	No	No	4
Yoo	2009	Minimal	No	No	No	No	No	No	No	0
		Internet	No	Yes	Yes	No	No	No	No	2

Note. Motivate=motivational interviewing; person_b = basic in-person intervention; person_e = enhanced in-person intervention; Internet_b = basic Internet-based intervention; Internet_e = enhanced Internet-based intervention. Minimal arm includes control, wait-list, usual care groups or the group that only received standard health information.
