# Supplementary material for: Evaluation of Internet-Based Interventions on Waist Circumference Reduction: A Meta-Analysis
Source: J Med Internet Res. 2015 Jul 21;17(7):e181. doi: 10.2196/jmir.3921 (PMC4527011; doi:10.2196/jmir.3921)
Supplement: Supplementary file 3 [file jmir_v17i7e181_app3.pdf]

Appendix 3: Table for the risk of bias assessment

Author	Year	Randomization		Blinding		Incomplete	Selective	Other	Overall
		Sequence	Allocation	Participants/Personnel	Measurements				
Bennett	2010	?	-	?	-	-	-	-	?
Bischoff	2010	?	?	?	?	+	-	+	+
Booth	2008	?	?	+	?	-	-	+	+
Bukhari	2009	-	-	?	?	-	-	+	+
Carr	2008	?	?	?	?	-	-	+	?
Chambliss	2011	-	-	?	?	-	-	-	?
Chen	2013	?	?	?	?	-	-	+	+
Chung	2014	-	-	+	-	-	-	-	+
Collins	2012	-	-	+	-	-	-	-	+
Dekkers	2011	-	-	+	+	-	-	+	+
Hansen	2012	?	?	+	+	-	-	-	+
Herrick	2009	-	-	?	?	-	-	-	?
Hunter	2008	-	?	+	?	-	-	+	+
Kang	2010	-	-	?	?	-	-	-	?

Note. - = low risk of bias, + = high risk of bias, ? = unclear on the risk of bias.
