# Supplementary material for: Evaluation of Internet-Based Interventions on Waist Circumference Reduction: A Meta-Analysis
Source: J Med Internet Res. 2015 Jul 21;17(7):e181. doi: 10.2196/jmir.3921 (PMC4527011; doi:10.2196/jmir.3921)
Supplement: Supplementary file 4 [file jmir_v17i7e181_app4.pdf]

Appendix 4: Funnel plot for publication bias

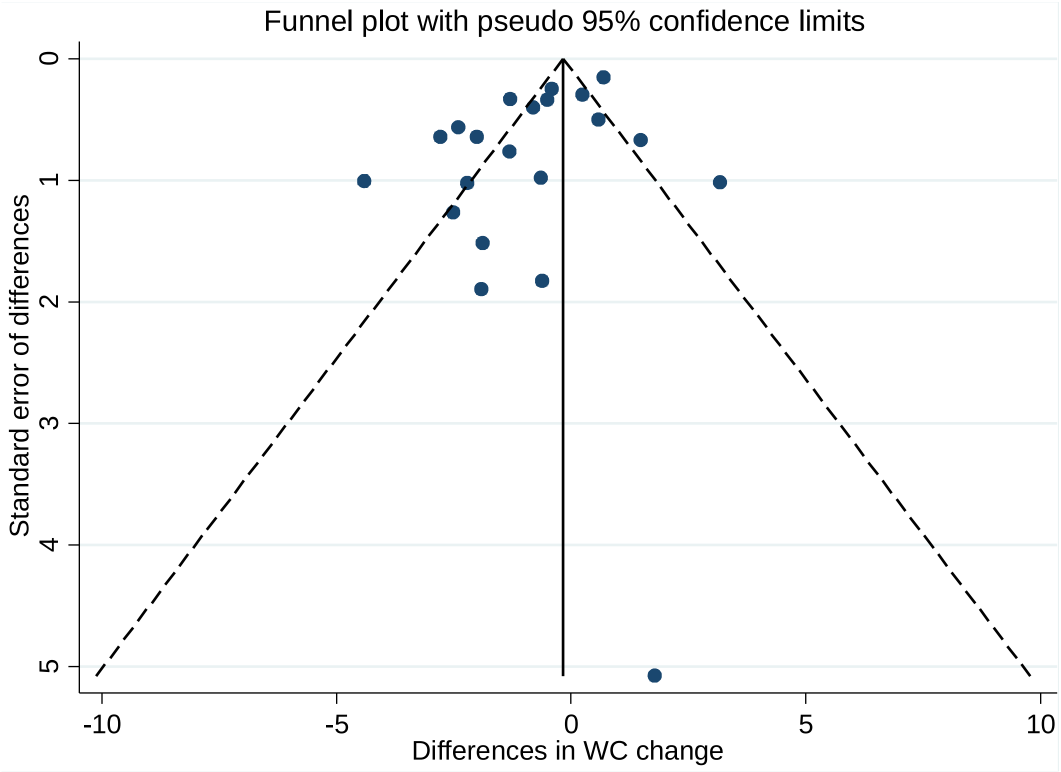

Note. This plot visualizes the waist circumference change in each study arm for all Internet-based interventions reviewed in this meta-analysis. It overall indicates absence of publication bias as the reviewed studies are distributed symmetrically about the mean effect size.
