# Supplementary material for: Evaluation of Internet-Based Interventions on Waist Circumference Reduction: A Meta-Analysis
Source: J Med Internet Res. 2015 Jul 21;17(7):e181. doi: 10.2196/jmir.3921 (PMC4527011; doi:10.2196/jmir.3921)
Supplement: Supplementary file 5 [file jmir_v17i7e181_app5.pdf]

Appendix 5: Content and supplementary approaches of sub-studies included in this meta-analysis

Author	Year	Arm	Content	Supplementary Approach
Bennett	2010	Minimal	Both	
		Internet	Both	Two in-person coaching + 2 phone coaching
Bischoff	2010	Internet_b	PA	Pedometer
		Internet_e	PA	Pedometer
Booth	2008	Internet_b	PA	Pedometer
		Internet_e	Both	Pedometer
Bukhari	2009	Minimal	Diet	
		Internet	Diet	
Carr	2008	Minimal	PA	
		Internet	PA	Pedometer
Chambliss	2011	Minimal	Both	
		Internet_b	Both	One in-person seminar
		Internet_e	Both	One in-person seminar + monthly brief phone consultations
Chen	2013	Minimal	Both	
		Internet	Both	
Chung	2014	Minimal	Diet	One in-person seminar + brochure
		Paper	Diet	One in-person seminar + brochure
		Internet	Diet	One in-person seminar + brochure
Collins	2012	Minimal	Both	
		Internet_b	Both	
		Internet_e	Both	Call reminders when necessary
Dekkers	2011	Minimal	Both	Brochure
		Phone	Both	Brochure
		Internet	Both	Brochure + SMS
Hansen	2012	Minimal	None	
		Internet	PA	
Herrick	2009	Minimal	Both	
		Internet	Both	

Hunter	2008	Minimal	Both	Annual health assessment
		Internet	Both	In-person orientation + 2 short phone calls
Kang	2010	Minimal	Both	
		Internet_b	Both	In-person counseling
		Internet_e	Both	In-person counseling
Mehring	2013	Minimal	Both	
		Internet	Both	Phone calls + SMS
Mobley	2006	Person_b	Both	One class
		Person_e	Both	One tailored counseling
		Internet_b	Both	One class
		Internet_e	Both	One tailored counseling
Morgan	2009	Minimal	Both	One in-person session + booklet
		Internet	Both	One in-person session + booklet + pedometer
Morgan	2011	Minimal	Both	
		Internet	Both	DVD + pedometer + tape
Morgan	2013	Minimal	Both	Book + DVD + pedometer + tape
		Paper	Both	
		Internet	Both	One in-person session + book + pedometer
Patrick	2011	Minimal	Both	
		Internet	Both	Pedometer
Pressler	2010	Internet_b	PA	One in-person session
		Internet_e	PA	One in-person session
Pullen	2008	Internet_b	Both	Pedometer
		Internet_e	Both	Pedometer
Rogers	2012	Paper	Both	
		Internet_b	Both	Wearable monitor + monthly call
		Internet_e	Both	Wearable monitor + monthly call
Seely	2013	Minimal	Both	One class
		Internet	Both	One class
Tate	2001	Internet_b	Both	One in-person group session

		Internet_e	Both	One in-person group session
Tate	2003	Internet_b	Both	One in-person group session
		Internet_e	Both	One in-person group session
van Genugten	2012	Internet_b	Both	
		Internet_e	Both	Phone call follow up when necessary
van Wier	2009	Minimal	Both	
		Internet	Both	Brochure + pedometer
		Phone	Both	Brochure + binder + pedometer
Webber	2010	Internet_b	Both	Two in-person sessions
		Internet_e	Both	Two in-person sessions
Wijsman	2013	Minimal	PA	
		Internet	PA	Accelerometer
Yoo	2009	Minimal	Both	Usual care
		Internet	Both	Usual care + Smart phone programs + SMS

Note. Person_b = basic in-person intervention; person_e = enhanced in-person intervention; Internet_b = basic Internet-based intervention; Internet_e = enhanced Internet-based intervention. PA = physical activity. SMS = short message service text messaging. “Both” refers to the intervention that addresses both physical activity and healthy diet. Minimal arm includes control, wait-list, usual care groups or the group that only received standard health information.
